# Dr. J. B. Drake

**Published:** 1916-08

**Authors:** 


**Dr. J. B. Drake,** P. & S., Baltimore, 1882, of Norwich, Chenango Co., died suddenly at the wheel of his automobile, July 12, the car running into the lobby of the Colonial Theatre but doing no material damage. He had practiced medicine in Norwich for 15 years. His age is stated at 50, which does not correspond with the date of his graduation in medicine.

